# Achieving universal coverage of childhood cancers in Ghana via the National Health Insurance Scheme: A stakeholder analysis

**DOI:** 10.1371/journal.pgph.0004871

**Published:** 2025-07-21

**Authors:** Richmond Owusu, Lieke Fleur Heupink, Godwin Gulbi, Brian Asare, Ivy Amankwah, Emmanuella Abassah-Konadu, Desmond Dzidzornu Otoo, Joycelyn Azeez, Martha Gyansa-Lutterodt, Lydia Dsane-Selby, Ruby Aileen Mensah, Saviour Yevutsey, William Omane-Adjekum, Francis Ruiz, Mohamed Gad, Justice Nonvignon, Lumbwe Chola, Ghana Health Technology Assessment Technical Working Group

**Affiliations:** 1 Department of Health Policy, Planning, and Management, University of Ghana School of Public Health, Accra, Ghana; 2 Norwegian Institute of Public Health, Oslo, Norway; 3 Ministry of Health, Accra, Ghana; 4 National Health Insurance Authority, Accra, Ghana; 5 London School of Hygiene & Tropical Medicine, London, United Kingdom; Federal University Birnin Kebbi, NIGERIA

## Abstract

Childhood cancers present a significant health problem and contribute to global child mortality. Low- and middle-income countries experience higher rates of childhood cancers with survival rates between 10% and 50%. In Ghana, about 2,500 children are diagnosed with cancer annually. Despite availability of effective management strategies, childhood cancers are not fully integrated into the NHIS, leaving patients and caregivers to make out-of-pocket payments leading to delayed diagnosis and treatment abandonment. Although stakeholders have made efforts to address the issue, the various stakeholders in childhood cancer management and their roles are still unclear. The study sought to identify and analyze stakeholders involved and challenges in childhood cancer management and financing in Ghana. A stakeholder analysis was conducted which included a rapid review of policy documents and a stakeholder engagement workshop. 21 stakeholders were purposively selected and focus group discussions were held with an interview guide at a one-day stakeholder engagement meeting. Stakeholders were categorized using Mendelow’s power-interest grid, and their roles, interests, and influence on childhood cancer policies were assessed. Key stakeholders identified included the Ministry of Health, NHIA, healthcare providers, NGOs, WHO, and patient advocacy groups. The Ministry of Health, NHIA, and healthcare providers were primary drivers with high interest and influence. The burden of Burkitt’s Lymphoma constituted 30–35% of all childhood cancer cases. Ghana has adapted treatment protocols with some inclusion on the NHIS. However, NHIS tariffs remain low. Challenges in managing Burkitt’s Lymphoma included inadequate reimbursement rates, high treatment costs, treatment abandonment, limited access to paediatric oncology specialists and indirect costs such as transportation and accommodation. Achieving universal health coverage through management and financing of childhood cancers in Ghana requires comprehensive policies, equitable financial coverage under the NHIS, enhanced stakeholder collaboration and increased investments in building capacity of paediatric oncologists in Ghana.

## Introduction

Childhood cancers are a major driver of mortality in children and adolescents worldwide [1–3] Among children under 14 years, approximately 397,000 cases of cancer are diagnosed every year with approximately 90% recorded in low-and-middle-income countries (LMICs) [4,5]. In high income countries (HICs), the survival rate of childhood cancers is over 80% [6], however in LMICs the survival rate in children with cancers are between 10% to 50% due to diverse health system challenges [7,8]. Thus, children with cancer in LMICs are four times more likely to die compared to their counterparts in HICs [9]. Treatment abandonment due to high cost of treatment, low level of diagnosis, and lack of specialized care are important contributors to these poor outcomes [9].

In Ghana, about 1200 children under the age of 15 years are estimated to develop cancer annually [10]. The current trend shows that there is an increase, probably because of improved diagnosis and early detection of cases. In 2018, the World Health Organization (WHO) launched the Global Initiative for Childhood Cancer with the goal of increasing childhood cancer survival globally to 60% by 2030 and save one million children with cancer in the next decade [9]. In response, countries, including Ghana, need to strengthen their health systems that are necessary for achieving this goal. For example, early detection, specialist care, and complete treatment without abandonment will be supported by strong health system which is well funded, with no financial barrier to care, and well-trained pediatric oncologists. Unfortunately, the Ghanaian health system faces a number of challenges with regards to childhood cancer management. Foremost, despite a reported evidence of childhood cancer management in Ghana being very cost-effective [7], it is not integrated into national social programs like the National Health Insurance Scheme (NHIS). Neither are all medicines necessary to treat childhood cancers part of the essential medicine list (EML). The relevant cancer medicines on the EML are not intended for treatments of childhood cancers, but often only included in the treatment of other cancers such as breast cancer. Worse yet, access to essential childhood cancer drugs is a challenge with a reported regular stock-out occurring for 88% of essential childhood cancer drugs with a 70-day median stock-out duration [11]. This leaves the young patients and their caregivers to pay for cancer care, including medicines, out-of-pocket, forming a barrier that often results in delayed diagnosis and treatment abandonment [9].

Ghana has a history of responding to key national health issues of interest. Given the effective management of childhood cancers is on global agenda and the fact that childhood cancers are a public health challenge in Ghana, the response of stakeholders in addressing it is vital. In Ghana, a number of policies are in place/aim to control the increasing prevalence of non-communicable diseases (NCDs) including childhood cancers. Some of the policies are focused on treatment and care (e.g., Essential Medicines List, Standard Treatment Guidelines) whereas others target prevention and control (e.g., National Policy for the Prevention and Control of NCDs in Ghana, Public Health Act). These existing policies are commendable and contribute to management of childhood cancers in Ghana. Notwithstanding this, the various stakeholders in the childhood cancer management and their roles are still unclear. Yet, stakeholder engagement is a necessary tool to successful development and implementation of health policies and guidelines [12–14].

The efforts of the government of Ghana to address the increasing problem of childhood cancers in the country requires that relevant stakeholders effectively contribute to this effort. Stakeholders have been defined as “*individuals, organizations or communities that have a direct interest in the process and outcomes of a project, research or policy endeavor*” [15](p. 5). In most cases, the stakeholder is either ‘affected by’ or ‘affects’ the policy in question. Concannon et al. conceptualizes this in a framework of 7Ps which includes: patients and the public, providers, purchasers, payers, public policymakers and policy advocates working in the non-governmental sector, product makers, and principal investigators [16]. In the light of policy analysis approaches, a stakeholder analysis can provide a conceptualization that assists in the analysis of interests and influence of the actors, their interrelations and impact on policy, within a broader political, economic and cultural context. With regards to childhood cancer management in Ghana, there is limited information about the stakeholders involved and their respective roles. Our literature search revealed that no study has targeted stakeholder analysis on childhood cancer in Ghana. Against this backdrop, we sought to conduct a stakeholder analysis for the purpose of identifying and mapping of stakeholders involved and challenges in childhood cancer management and financing in Ghana. This was aimed at eliciting relevant evidence that will contribute to management of childhood cancers in Ghana.

## Materials and methods

### Study design

The study design is primarily a stakeholder analysis, which is defined as “a process of systematically gathering and analyzing qualitative information to determine whose interests should be considered when developing and/or implementing a policy or program’ [17].

### Setting

In Ghana, there are over 11.5 million children that are aged 0 – 14 years, this represents more than one-third of the country’s total population [18]. Ghana has progressively improved its health system and child health generally. In 2003, the NHIS was introduced as a public health care financing mechanism to improve healthcare access by removing any financial barrier to care while providing financial risk protection for the residents in Ghana [19]. It is reported that about 95% of disease conditions in Ghana is covered by the NHIS [20]. The healthcare system of Ghana is hierarchical, ranging from the lowest level – community, sub-district, district, regional and national. In this, primary, secondary, and tertiary care occur in various healthcare facilities that are distributed across these levels. While primary care facilities in the communities play an important role in the early diagnosis and referral of patients to these specialist facilities, teaching hospitals for example provide specialist/tertiary care at the regional and national levels. There are five Teaching hospitals in Ghana, however, currently Korle-Bu and Komfo Anokye teaching hospitals remain the main providers of cancer care for children.

### Sampling and data collection

We used two main approaches for this stakeholder analysis. First, we relied on a rapid literature review of policy documents in Ghana that are related to childhood cancer management/treatment either in part or whole. Further, we searched webpages of identified stakeholders to obtain additional information about their functions and characteristics. In essence, we used existing policy documents for this stakeholder analysis alongside primary data from the stakeholder engagement.

Secondly, for primary data we engaged representatives of organizations who have a substantive stake in any change in childhood cancer management and financing in Ghana, in that they will either affect its design and/or implementation or be affected by it [21]. In this, sampling was done purposively through discussion with the Ministry of Health. Due to the COVID-19 situation, a one-day stakeholder engagement workshop was organized by the Health Technology Assessment (HTA) secretariat of Ministry of Health where participation was in-person and online. Zoom video conferencing platform was used by participants who could not join the meeting in-person. The HTA secretariat invited identified key actors for childhood cancer management and financing in Ghana for the participation in the stakeholder engagement workshop. A focus group discussion approach was used in the engagement of participants, with an interview guide that provided structure and flow of the discussion. Notwithstanding this, enough flexibility was allowed to include any information that was not planned for in the guide but was relevant in the discussion for consideration.

### Analysis

**Fig 1:**
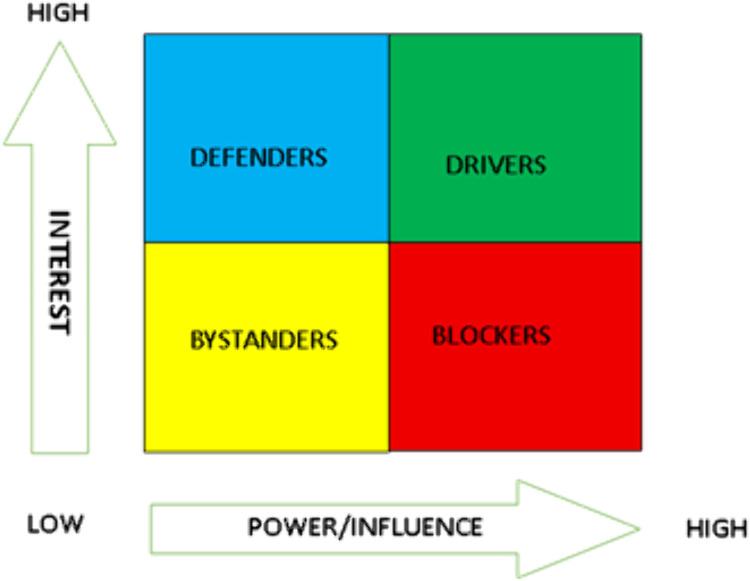
Mendelow’s power-interest grid. Source: McMullan et al. [23].

We divided stakeholders into 4 categories: pharmaceutical sector, public health, government, non-governmental organizations (NGOs) and research organizations. Using existing framework, we examined the interplay of power/influence and interest to rank stakeholders. The level of power/influence was explored in the context of alliance, legal backing, and resources of stakeholders [17,21,22]. Actors’ ‘influence/power’ was grouped as low, medium, or high on policies concerning childhood cancers and financing in Ghana. Similarly, ‘interest’ of actors was categorized as low, medium, or high in policies regarding childhood cancer management and financing in Ghana. Thus, to establish a stakeholder mapping we used Mendelow’s power-interest grid for analysis (Fig 1). Each stakeholder was assessed and was classified into one of the following categories: a ‘defender’ (high interest, low power), ‘bystander’ (low interest, low power), ‘driver’ (high interest, high power) or ‘blocker’ (low interest, high power) [23].

### Ethical considerations

This work is a product of a bigger Health Technology Assessment on Burkitt Lymphoma/childhood cancer coverage under the National Health Insurance Scheme which relied on secondary data and did not require ethical approval. In this stakeholder analysis work, informed consent was obtained from workshop participants. A waiver was obtained from the University of Ghana Medical Center Institutional Review Board (UGMC/IRBREVIEW/093/21). Also, the entirety of the gathered data was rendered anonymous.

## Results

### Identification of stakeholders

The review of policy documents in Ghana resulted in four main policy documents for Childhood Cancers: Standard Treatment Guidelines, Essential Medicines List, NHIS Essential Medicines List, National Health Insurance Act, 2012 (Act 852). These documents gave insight into the relevant stakeholders for childhood cancer management in Ghana. The main actors/ stakeholders for Childhood cancer in Ghana were found to be the Ministry of Health, Healthcare providers in Teaching hospitals, Ghana Health Service (GHS), Christian Health Association of Ghana (CHAG), private providers, quasi-government, the National Health Insurance Authority (NHIA), Patient groups, NGOs and WHO. (Table 1).

**Table 1:** Stakeholders identified and their primary role in childhood cancer management.

Stakeholder	Role
Ministry of Health	Policy Formulation
Healthcare providers (i.e., GHS, Teaching hospitals, CHAG, private, Quasi-government, GHS facilities)	Service delivery and Implementation of Policies, Prevention and health promotion treatment and management of diabetes
National Health Insurance Authority	Financing
International organisations (WHO/ PATH Health partners)	Technical support
Patient group (Childhood Cancer Society of Ghana)	Educate, advocate for and provide support services for patients and their caregivers.
NGOs for Childhood Cancer (e.g., Lifeline for Childhood Cancer Foundation Ghana)	Advocacy

### Power-interest analysis of identified stakeholders

Using Mendelow’s power-interest grid for stakeholder analysis, stakeholders identified via literature review were categorized as drivers, defenders, bystanders, or blockers. This was based on their varying interests and power/influence with regards to management and financing of childhood cancers in Ghana.

The Ministry of Health, NHIA, and healthcare providers were the main stakeholders identified as drivers, having high interest and high power/influence with regards to guidelines/policies relating to childhood cancers. Defenders for childhood cancer were found to be Childhood Cancer Society of Ghana and the WHO. Defenders have high interests in policies however their power/influence remains low. From the review, the bystanders were found to be NGOs for Childhood cancers. Though these groups of people have an interest in all policies regarding Childhood cancer, the level of interest is relatively low, as well as their ability and power to influence. From the stakeholders identified, none fell under the blockers category.

### Results of stakeholder engagement

Table 2 below shows the number of stakeholders who were engaged in Childhood cancer management and financing in Ghana.

**Table 2:** Stakeholders Engaged on Management and Financing of Childhood Cancer, 2021.

Stakeholders	Number of Participants (N = 21)
Ministry of Health	9
Healthcare Providers	4
NHIA WHO	3 2
Research/ Academic institutions Patient Groups	2 –
NGOs	1

The results from the stakeholder engagement were captured under five main thematic areas:

- Burden of Burkitt’s Lymphoma (BL)
- Management of Burkitt’s Lymphoma
- Financing of Burkitt’s Lymphoma
- Challenges in management of Burkitt’s Lymphoma
- Policies on Burkitt’s Lymphoma in Ghana

### Burden of Burkitt’s Lymphoma

From the stakeholder engagement, it was revealed that Lymphoma constitutes 30–35% of all childhood cancer cases in children. Amongst childhood cancers, the incidence of leukemia is also increasing. Lymphomas can be deadly if not treated. BL consists of the endemic type, which is located in the jaw as well as the sporadic type. In recent times, more cases of the sporadic type are emerging. Lymphomas can be aggressive if not treated early and further treatment delays, the more expensive treatment is once it is commenced. Many children have survived after obtaining adequate treatment; however, risk depends on how advanced the disease is. There are three categories: local, locally advanced and static disease in bone marrow or brain. The static disease in bone marrow or brain is associated with a high risk of relapse. Whenever patients progress to the advanced disease (metastatic cancer), there is the need to provide more aggressive treatment to lower risk of relapse, however locally advanced cancers may also require aggressive treatment. A stakeholder indicated that *“if disease diagnosed in stage III is locally advanced and not metastatic, aggressive treatment should lower the metastatic burden.”*

It was also indicated that approximately 2500 children were diagnosed with childhood cancers each year. In Ghana, there are currently 7 paediatric oncologists for the entire population (30 million), however practitioners with other specialties (such as haematologists and standard oncologists) treat BL in Ghana.

### Management of Burkitt’s Lymphoma

The engagement provided insight on management of BL in Ghana. A stakeholder mentioned that currently, Ghana is using an adapted version treatment protocol which fits the country context*. “For example, a “high” dose of methotrexate is still lower in Ghana than in the West, treatment is there to prevent relapse, treat child, while not emptying the pockets…”*

It was stated that even though Methotrexate can be given intramuscularly (as used in the Health Technology Assessment (HTA) analysis on BL), when being given in high doses, an intravenous route is appropriate. IV methotrexate is usually added in Stage IV of BL. The interval between the courses is two weeks, and to prevent patients from becoming resistant, several drugs are used. The medications are given alternatively and are given up to eight cycles. Treatment can be extended depending on the advancement of the disease.

Aside medications, supportive care is also provided. This tends to be more expensive than the cancer treatment itself. Nutrition is a key component in management and food provided on admission does not come at an extra cost, however it is sometimes necessary to engage Dieticians, and patients are sometimes unable to afford such services.

### Financing of Burkitt’s Lymphoma

As a response to reasons why the cost of treatment on NHIS is lower than the cost of treatment without NHIS coverage, it was stated that it depends on the perspective being considered. The NHIS tariffs will never equate to the cost charged by the healthcare provider due to reasons such as wastage. In addition, some diagnostics are not covered by the NHIS. There is no value for unit cost, such as diagnostic tests, and in such case, user fees are used as proxy for the unit costs. The NHIS focuses on results from budget impact analysis, as this plays a key role in reimbursement decisions rather than results from cost-effectiveness analysis.

### Challenges in management of Burkitt’s Lymphoma

#### Reimbursement challenges.

According to the stakeholders, the NHIS reimbursement rate is too low, and this has led to hospitals charging for services. The goal should be to have the BL management of the child fully reimbursed. Many patients are covered by the NHIS but unfortunately are still paying.

The NHIS currently does not fully cover immunotherapies, but some of the treatments are already on the Essential Medicine List. A good course of action will be to fully verify how diagnosis is made, how much treatment of BL costs, how many courses are needed, intervals between courses, when patients are in remission, etc. This will give a comprehensive idea of the total reimbursement cost to the NHIA, thereby reducing gaps that allow for co-payment.

#### Cost of treatment.

It was indicated that some patients have no money for treatment, and they depend on goodwill from individuals. It is not just chemotherapy, which is expensive, other costs to be considered are transportation to treatment centres and accommodation. There are situations where parents have to stop working to look after the sick child, who may be just one of many children. When medication costs are covered, uptake of treatment will increase, however, individuals have to be ready for the additional cost that have to be paid.

#### Abandonment/ disrupted treatment.

Abandonment of treatment is due to various reasons such as cost and accessibility of health facility and affects prognosis of the disease. A typical example was given by a stakeholder, who said “*I have a child who I discovered with Burkitt’s in Ashanti who was being treated at Agogo Hospital. I hear she has absconded once the tumour size went down after about 6 months of treatment. If we get her back, what is the prognosis now?”.*

There are challenges with traditional medicine usage in BL. Patients get assurance from herbal practitioners that they can be cured, leading them to abandon treatment at health facilities. When some patients are scheduled to come back to continue treatment, they never show up. There is the need to ensure that patients come back to treatment centre, even when treatment is not possible, palliative care should be provided.

There is currently no indication on abandonment rate. Estimates have been used based on studies. If patients are fully covered, abandonment rate can reduce up to 80%. This assumption was made by a stakeholder present.

A healthcare practitioner indicated that they could share current data on abandonment rate, and that abandonment rate was now less than 10% due to the involvement of NGOs in the management of BL. There was a plea to fully investigate the reasons for treatment abandonment.

### Policies on Burkitt’s Lymphoma in Ghana

There is the need for policies that look at holistic care of BL patients, including supportive care such as nutrition during treatment. Policies focusing on palliative care of BL should also be considered. For policy purposes it will be beneficial to assess the scenarios for different settings or system perspective; tertiary, private, other settings when it comes to treatment of BL.

## Discussion

The Stakeholder analysis allowed for identification and exploration of interests and influence of various stakeholders in the management and financing of childhood cancers in Ghana.

The analysis identified the Ministry of Health as a major driver of childhood cancers, with very high interest and high influence, responsible for enacting policies on childhood cancer management in Ghana. This includes the development of treatment guidelines and essential medicines list for childhood cancers. The 2017 Standard Treatment Guideline (STG) provides guidelines for treating both Hodgkin’s Lymphoma, non-Hodgkin’s Lymphoma (e.g. Burkitt’s Lymphoma), as well as Wilms Tumour and Retinoblastoma. These childhood cancers are prevalent in Ghana, however guidelines for the management of childhood cancers such as Neuroblastoma and Leukemia are absent, thereby presenting a gap. There is a strategy document for Cancer Control in Ghana (2012 – 2016) which broadly addresses all cancers and mentions childhood cancers in Ghana, highlighting strategies such as general awareness creation on the signs and symptoms of childhood cancer to encourage early detection and training of health care workers to institute newborn checks for signs of some childhood cancers [24].

Another major driver of Childhood Cancers, with very high power and interest in Ghana, is the NHIA. The NHIA oversees the provision of equitable access and financial coverage for health services, including management of childhood cancers, through the National Health Insurance Scheme (NHIS). The significant barrier to access essential cancer medicines in LMICs, including Ghana, is the issue of high prices. In December 2021, the NHIA announced the inclusion of four childhood cancers as part of their benefits package [25], thus reducing the huge financial burden that comes with seeking and receiving care for childhood cancers. These include Neuroblastoma (childhood cancer of the jaw and abdomen), Leukaemia (childhood cancer of the blood), Retinoblastoma (childhood cancer of the eye) and Wilms Tumour (childhood cancer of the kidney).

Healthcare providers also have a high interest and power/ influence with regards to childhood cancer management in Ghana. Their interest and power stem from the fact that they are mandated to provide clinical care for children with cancer. Ghana has two main childhood cancer treatment centres: Korle Bu Teaching Hospital (KBTH) and Komfo Anokye Teaching Hospital (KATH), that are responsible for offering comprehensive childhood cancer services. With only two major cancer treatment centers in Ghana, access to childhood cancer care presents a major challenge to patients, especially those in rural areas. There are also only seven trained pediatric oncologists in Ghana as of 2023 [26] for a population of over 30 million [27] and childhood cancer care may be delayed owing to this limited medical capacity.

Patient groups and international organizations such as the WHO are defenders of childhood cancer, having a high interest yet low power to influence childhood cancer policies and care. Though they have low power, they contribute to effective management of childhood cancers. Ghana Parents Association for Childhood Cancers (GHAPACC) is an association of parents of children diagnosed with cancers and other life- threatening blood disorders, health professionals involved in caring for such children and survivors of childhood cancers. An extension of patient groups was also considered under this analysis. This group, known as the Childhood Cancer Society of Ghana (CCSG), is a professional society of health workers and other stakeholders who are directly involved in the management of children with cancers. This society aims to ensure early detection and unimpeded access to quality care throughout the continuum of life for affected children and their families, and its establishment was facilitated by the Korle Bu Teaching Hospital and Komfo Anokye Teaching Hospital. As an advocacy group, they ensure affected children can access effective quality lifesaving care wherever they are in the country.

The analysis revealed NGOs to have relatively low interest and power/ influence and the need to boost their influence/ power. NGOs play a critical role in addressing issues that may be overlooked by government and industry, help raise knowledge and awareness of childhood cancers and advocate for policy change and enforcement [28]. In Ghana, one of the major NGOs for childhood cancer is Lifeline for Childhood Cancer, whose mission is to optimize access to the best possible care for all children affected by cancer in Ghana, ensuring that no child suffers needlessly. The influence of NGOs can be increased through encouraging sustainable partnerships with agencies such as the Ministry of Health and having reliable and active communication platforms/forums for effective discourse.

Engaging with stakeholders through focus group discussions also allowed for insight and exploration of stakeholder perspectives on childhood cancers, using Burkitt’s Lymphoma as a proxy. Perspectives on the incidence and prevalence of childhood cancers (Burkitt’s Lymphoma), the types and stages of cancers, the need for early treatment, treatment options, abandonment rates and the need for supportive care during and after treatment of childhood cancers were discussed. Other areas that were explored included the indirect catastrophic costs of cancer to the family, reimbursement of childhood cancer management as well as policy issues regarding childhood cancers in Ghana.

According to the World Health Organization (WHO), there is a global lack of approximately 4.3 million healthcare professionals, primarily in South Asia and Africa and these regions also have the highest burden of illness, exacerbated by a smaller healthcare workforce [29]. It is stated that individuals in LMICs are deprived of sufficient levels of healthcare and cannot access medical facilities in a timely manner [30]. For childhood cancers, the discussions with stakeholders revealed a relatively high burden of childhood cancers (2500 children per year), with limited number of health facilities and oncologists in the country. As stakeholders with high power and influence, especially in management of childhood cancers, an increase in healthcare providers and resources to function will be critical in reducing the burden of childhood cancers.

The NHIA plays a financing role and has a high interest and power when it comes to childhood cancers. The discussions from the engagement focused on the difference in reimbursement rates for childhood cancers by the NHIA and healthcare providers. The NHIA emphasized the perspective taken in computation of costs as the key reason for the difference in costs, highlighting that a societal perspective taken will always incur more costs. Another key reason highlighted was the issue of wastage, which makes healthcare provider cost values higher than NHIA costs. Although defined narrowly as “inefficient and wasteful spending,” medical waste encompasses a wide range of complex and interrelated issues, which includes clinical inefficiencies, overuse, as well as excessive prices, and leads to staggering costs [31].

The stakeholder engagement also highlighted the key challenges in managing childhood cancers which were broadly classified as reimbursement challenges, cost of treatment and abandonment/disrupted treatment. The inclusion of four childhood cancers as part of the NHIA benefits package [25] will reduce reimbursement challenges as well as financial cost to patients. Treatment abandonment is recognized as a leading cause of treatment failure for children with cancer in low-and-middle-income countries (LMIC) [32,33]. To increase pediatric cancer survival rate in LMICs, cancer-related deaths must be decreased not just by promoting early diagnosis, delivering effective care, and reducing treatment-related death, but also by reducing treatment abandonment [32].

Efforts to improve early detection and treatment of diseases through improved access to high-quality medical care must be complemented by other approaches, such as policy formulation, to put in place decisions, plans and actions required to achieve specific health goals. The Ministry of Health plays an active role in enacting policies and guidelines for various diseases. Although there are guidelines for treatment of some childhood cancers in Ghana, other cancers such as Neuroblastoma and Leukemia are not covered. Stakeholders agreed on the need for more specific policies on childhood cancers that consider a more comprehensive approach to management of childhood cancers, including supportive and palliative care.

## Conclusion

Stakeholder played different roles with different level of interests, and influence in the management and financing of childhood cancers in Ghana. Key stakeholders such as the Ministry of Health, NHIA, and healthcare providers were critical drivers showing both high interest and influence in addressing childhood cancers.

Though the burden of Burkitt’s lymphoma was found to be between 30–35% of all childhood cancers, significant challenges in management and finance include insufficient NHIS reimbursement, high treatment costs, and treatment abandonment. These challenges are exacerbated by the limited number of paediatric oncology specialists in Ghana and the high indirect costs of treatment, such as transportation, accommodation and cost of nutrition or supportive care.

Although progress has been made with the inclusion of some childhood cancers in the NHIS benefits package, the scheme does not provide comprehensive financial coverage and supportive care. To improve access to care for paediatric cancers in Ghana, policies and guidelines must be put in place to ensure early diagnosis, affordable and accessible treatment and comprehensive care coverage including supportive care. The human resource capacity can be strengthened through targeted investments in training more pediatric oncologists with equitable distribution across the country. Fostering partnerships and stakeholder collaborations can reduce the high economic and social burden of childhood cancers, significantly reduce treatment abandonment, foster equitable access to care, and ultimately improve survival rates for children with cancer in Ghana.
